# The first detection of *Echinococcus multilocularis* DNA in environmental fruit, vegetable, and mushroom samples using nested PCR

**DOI:** 10.1007/s00436-015-4630-9

**Published:** 2015-07-25

**Authors:** Anna Lass, Beata Szostakowska, Przemysław Myjak, Krzysztof Korzeniewski

**Affiliations:** Department of Tropical Parasitology, Institute of Maritime and Tropical Medicine in Gdynia, Medical University of Gdansk, 9b Powstania Styczniowego Str., 81-519 Gdynia, Poland; Epidemiology and Tropical Medicine Department in Gdynia, Military Institute of Medicine in Warsaw, Grudzińskiego St. 4, 81-103 Gdynia, Poland

**Keywords:** *Echinococcus multiloccularis*, Eggs, Environment, PCR, Fruits, Vegetables, Mushrooms

## Abstract

The aim of this study was to estimate the presence of *Echinococcus multilocularis* DNA in fruits, vegetables, and mushrooms in rural areas of Varmia-Masuria Province, Poland, which is the region with the highest number of human alveolar echinococcosis (AE) cases in this country. Recovery tests showed that *E. multilocularis* DNA is detectable in samples contaminated with at least 100 eggs of this tapeworm. In total, 103 environmental fruit, vegetable, and mushroom samples collected in forests, plantations, and kitchen gardens were analyzed using nested PCR assay based on the mitochondrial 12S ribosomal RNA (rRNA) gene. The parasite DNA was detected in 23.3 % of the samples. Sequencing confirmed that the obtained PCR products represented *E. multilocularis*. This study is the first environmental survey of the presence of *E. multilocularis* DNA in fruits, vegetables, and mushrooms intended for consumption. The results clearly demonstrate that it may be a direct source of human infections and shows the need to educate the public about the threat, especially people living in at-risk areas.

## Introduction

*Echinococcus multilocularis* is a cestode species of the genus *Echinococcus* and may cause alveolar echinococcosis (AE) being one of the most dangerous parasitic zoonosis with a high fatality rate reaching 50–70 %. The causative agent of the disease is the larval stage of the tapeworm which develops mainly in the liver and is characterized by numerous tumor-like vesicles that invade and destroy surrounding tissue. In some cases, metacestodes may spread from the liver to different organs, i.e., lungs, brain, or bones. The incubation time of the disease can vary between less than 5 and up to 15 years, and the initial phase is always asymptomatic (Amman and Eckert 1996). The number of AE cases is increasing worldwide. The annual morbidity rate in Europe equals from 0.02 to 1.4 cases per 100,000 habitats but may rise up to 11–40 cases in highly endemic areas.

In Poland, alveococcosis, known for single casuistic cases up to the 1970s, has shown an increasing morbidity and mortality rate since 1990. Nowadays, Poland is the fourth European country to have a number of AE cases higher than 120 (according to data recorded until 2011) (Głuszcz and Kałczak 1960; Wesołowski et al. 1970; Kern et al. 2003; Nahorski et al. 2013).

Transmission of *E. multilocularis* occurs predominately during the sylvatic cycle. Definitive hosts are some carnivores: mainly foxes but also other wild canids (coyotes, raccoon dogs, wolves) or wild felids. However, in some areas, also domestic dogs or cats may play a role as a part of synanthropic cycle. Many species of small mammals may serve as intermediate hosts for *E. multilocularis* although the most important are small rodents belonging to the family Arvicolinae and Cricetidae. Humans belong to aberrant (nonspecific) hosts of the tapeworm (Thiess et al. 2001; Machnicka-Rowińska et al. 2002; Romig 2003; Eckert and Deplazes 2004).

*E. multilocularis* is widely distributed around the Northern Hemisphere including Europe, northern, and central Eurasia and parts of Northern America. Recent studies in Europe and Asia have shown that the endemic area of *E. multilocularis* is larger than previously suspected and an invasion among foxes has regionally expanded from rural to urban areas (Deplazes et al. 2004; Eckert and Deplazes 2004). In Europe, the red fox plays a role as the main definitive host of this tapeworm and the migration of red foxes influence the parasite spreading (Eckert and Deplazes 2004; Karamon et al. 2014). In Poland, *E. multilocularis* was detected in the fox population for the first time in 1994 in the northern area of the country (Malczewski et al. 1995). Subsequent studies performed in different parts of the country showed not only the presence but also a distinct and dynamic increase in both the fox population (about four times) and the percentage of infected foxes (over three times) during the past 15–20 years (the same situation is observed in other endemic regions of Europe (Karamon et al. 2014). The highest level of *E. multilocularis* prevalence (50 %) was observed in Varmia-Masuria Province (northeast of Poland) in which the highest number of human AE cases was noted—67 of 123 totally recorded in Poland between 1990 and 2011 (Nahorski et al. 2013; Karamon et al. 2014).

People may be infected by the ingestion of *E. multilocularis* eggs excreted with the definitive host feces. Eggs may persist preserving their infectivity for about a year in favorable environmental conditions. They are somewhat resistant to low temperatures but susceptible to high temperature or drying. It is generally estimated that humans may be exposed to tapeworm eggs via contact with infected animals or contaminated food or environment. However, the significance of the various potential routes of transmission remain unknown (Eckert and Deplazes 2004). So far, the data about the distribution of *E. multilocularis* eggs in the environment is insufficient. A study concerning the occurrence of the eggs in soil were performed in Japan with microscopic investigation (Matsudo et al. 2003) and in Poland using molecular methods (Szostakowska et al. 2014). There is no data about the contamination of fruits or vegetables with *E. multilocularis* eggs despite of the fact that it is regarded as one of the most important transmission routes.

The aim of the study was the establishment of a recovery test of *E. multilocularis* eggs followed by detection of *E. multilocularis* DNA in the Varmia-Masuria Province environment including samples collected from forests (berries, mushrooms), plantations (fruits), and kitchen gardens (vegetables) and assessment of the degree of contamination. Consequently, results were to demonstrate that plants and fungi contaminated with *E. multilocularis* eggs may be a source of infection in humans.

## Material and methods

### Sampling

**Fig. 1:**
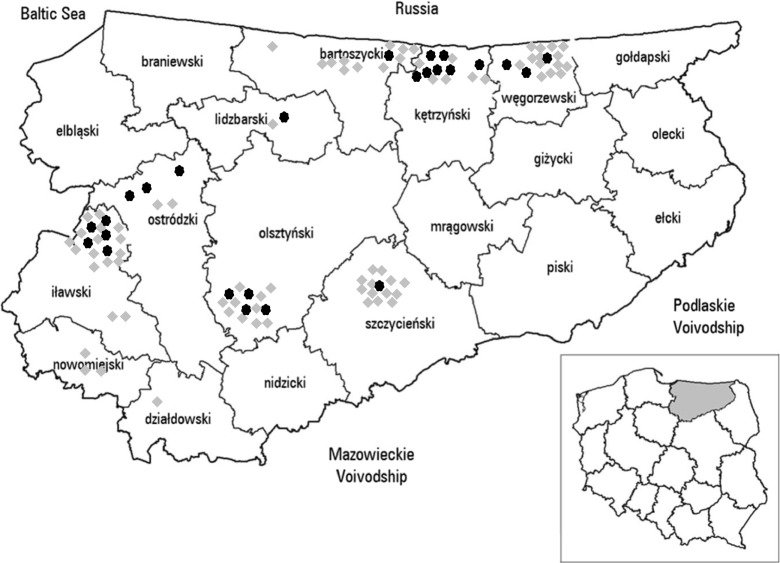
Origin fruit, vegetable, and mushroom samples analyzed in particular districts of Varmia-Masuria Province, Poland. *Gray dots* represent collected samples, and *black dots* represent positive samples

**Fig. 2:**
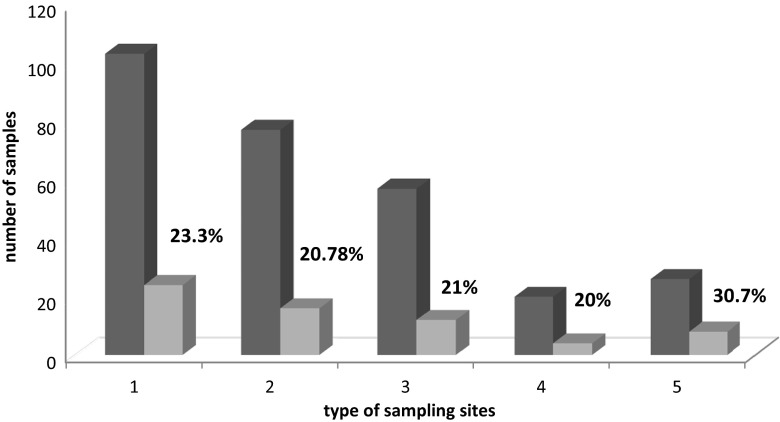
Comparison of positive results obtained from sampling sites around and aware from homesteads. *1* Total results for all sampling sites, *2* total results for sampling sites located away from homes (3 + 4), *3* results for sampling sites in forests, *4* results for sampling sites in plantations, *5* results for sampling sites close to homes

A total of 103 fruit, vegetable, and mushroom samples were collected between June 2011 and September 2012 at different sites located in ten districts of the Varmia-Masuria Province, northeastern Poland, where the highest number of human AE cases have been recorded (Nahorski et al. 2013). They originated from forests (*n* = 57), kitchen gardens (*n* = 26), and plantations (*n* = 20) (Table 1; Fig. 1). One sample consisted of 0.3–0.5 kg of fruits (raspberries, cranberries, blueberries, cowberries) or mushrooms, 0.5 kg of vegetables (carrot, parsley, beets, celery, radishes), or one lettuce or two bunches of dill or chives. The distance of sampling sites from homesteads was as follows: up to 50 m in case of kitchen gardens, from a few to several kilometers in case of forests, and over several kilometers in case of plantations (Fig. 2). The samples were put into disposable bags and transported to the laboratory.

**Table 1:** Detection of *Echinococcus multilocularis* DNA in environmental fruit, vegetable, and mushroom samples collected in the Varmia-Masuria Province, Poland in comparison to percent of infected foxes and number of human AE cases

Type of sampling site	Sample type	Number of samples tested	Positive samples		Number of human AE cases 1990–2011 (Nahorski et al. 2013)	% of infected foxes	
			Number %			2001–2006 (Malczewski et al. 2008)	2009–2013 (Karamon et al. 2014)
Forests	All undergrowth	57	12	21			
	Mushrooms	25	9	36			
	Forest fruits	32	3	9.4			
Kitchen gardens	Vegetables	26	8	30.7			
Plantations	Raspberries	20	4	20			
Total		103	24	23.3	65	39.6	50

### Recovery test form fruit, vegetable, and mushroom samples

Adult *E. multilocularis* tapeworms, which were stored in 70 % ethanol and used in all of the recovery experiments, were obtained from the National Veterinary Research Institute in Puławy, Poland. The DNA isolated from whole tapeworms was used as a positive control in the PCR experiments. Eggs obtained from gravid proglottids were used to estimate the efficiency of egg recovery from fruit, vegetable, and mushroom samples.

Initial experimental recovery tests were performed using suspensions of *E. multilocularis* eggs in distilled water, before evaluating the detection of *E. multilocularis* eggs in environmental samples. Series of egg-free fruit (raspberries), vegetable (radishes), and mushroom samples bought in greengrocers were washed in the laboratory with Tween 80 in order to minimize the risk of accidental contamination with *E. multilocularis*. The samples prepared in this way were experimentally contaminated with 10, 10^2^, and 10^3^ eggs. Then, egg recovery and specific detection of *E. multilocularis* DNA were performed as described below.

### Concentration of eggs

In order to concentrate and recover *E. multilocularis* eggs from collected samples, the flotation method using saturated ZnCl_2_ solution (density, 1.4 g/cm^3^) was employed. Briefly, one sample of fruit, vegetables, or mushrooms was placed in a glass vessel, mixed slightly with 2 l 0.05 % Tween 80 solution on an automatic orbital shaker (Multi PSU-20, BioSan, Warren, MI, USA) for 30 min at 120 rpm. Then, washings were transferred to another vessel while 0.5 l of 0.05 % Tween 80 solution was added again to the first vessel and mixed on an automatic orbital shaker for 5 min under the same conditions. Next, plants or mushrooms were removed; both washing fractions were mixed and left overnight (without shaking) at 4 °C. The next day, the supernatant was removed using an automatic pipette filler (Hirschmann Laborgeräte, Germany), and the remaining sediment (amount of about 100 ml) was filtered by set of sieves (pore size of the last sieve was 50 μm), placed in a 200-ml conical tube and centrifuged for 15 min at 200*×g*. The supernatant was removed, and the pellet obtained was placed in a 50-ml Falcon tube and frozen at −70 °C. Then, it was suspended in 30 ml of saturated ZnCl_2_ solution, mixed thoroughly, and centrifuged for 3 min at 200×*g*. Then, 20 ml of saturated ZnCl_2_ solution was added and centrifuged for 3 min at 200×*g*. Next, each tube was placed in a stand and topped up carefully with saturated ZnCl_2_ solution to form a positive meniscus. The surface of the liquid was covered with a cover glass slide for 15 min. Finally, the slide was washed with distilled water, and the material was retrieved in a 2.0-ml Eppendorf tube. To concentrate the tube content, it was centrifuged for 1 min at 200*×g*, and any excess water was removed carefully. Finally, the suspension obtained was preserved at −20 °C for further analysis.

### Molecular analysis

#### DNA extraction

Before DNA extraction, the material prepared from plants and mushroom samples was three times frozen at –70 °C and thawed at 30 °C in a water bath to destroy the egg walls and improve the efficiency of DNA extraction. DNA extraction was performed using a Sherlock AX Kit (A&A Biotechnology, Gdynia, Poland) according to the manufacturer’s instructions. All of the PCR templates were treated with an Anty-Inhibitor Kit (A&A Biotechnology, Gdynia, Poland) which removes polyphenolic PCR inhibitors using specific absorption particles, thereby removing factors that could interfere with the PCR. The PCR templates were stored at −20 °C.

#### Specific detection of *Echinococcus multilocularis* by nested PCR

The mitochondrial 12S ribosomal RNA (rRNA) gene was subjected to analysis using nested PCR reaction. The first step was performed using the primers p60for (5′-TTAAGATATATGTGGTACAGGATTAGATACCC-3′) and p375rev (5′-AACCGAGGGTGACGGGCGGTGTGTACC-3′) (von Nickisch-Rosenegk et al. 1999). The PCR amplification was performed in a 25 μl reaction mixture, which contained 2.5 μl 10× GeneAmp PCR Buffer II (Applied Biosystems, USA), 2 mM MgCl_2_ (Applied Biosystems, USA), 0.2 mM of each dNTP (Fermentas, Lithuania), 0.25 μM of each primer (Metabion, Germany), 1.25 U of AmpliTaq Gold polymerase (Applied Biosystems, USA), and 2 μl of DNA template. The amplification was performed according to the protocol described by Myjak et al. (2003) with one difference, i.e., an initial denaturation step for 15 min at 95 °C (conditions for AmpliTaq Gold polymerase). All of the products obtained in the first PCR step were diluted 1:10 with distilled water. The second step PCR reactions were performed using primers Em.nest/for (5′-GTGAGTGATTCTTGTTAGGGGAAGA-3′) and Em.nest/rev (5′-ACAATACCATATTACAACAATATTCCTATC-3′) (Dinkel et al. 1998; Dyachenko et al. 2008a) in a 25 μl reaction volume according to the protocol described by Dyachenko et al. (2008b) also preserving initial denaturation conditions proper for AmpliTaq Gold polymerase. The DNA isolated from adult *E. multilocularis* as a positive control and distilled water instead of a DNA template as a negative control were included in all of the PCR runs. All of the negative samples were examined for the presence of PCR inhibitors by mixing the DNA template isolated from examined environmental samples with the positive control. The PCR amplifications were performed using a GeneAmp PCR System 9700 Thermal Cycler (Applied Biosystems, USA). The PCR products were analyzed using a Gel Doc-It Imaging System (UVP, USA) after electrophoresis on a 2 % gel agarose, which was stained with Midori Green DNA Stain (Nippon Genetics Europe GmbH, Germany).

#### Sequencing

The final PCR products of the chosen positive samples were sequenced. Before sequencing PCR products were cleaned with the Clean Up Kit (A&A Biotechnology, Poland). The products of sequencing reaction, performed with the amplification primers, were cleaned with ExTerminator Kit (A&A Biotechnology, Poland) and subjected to analysis on an automatic sequencer AbiPrism 310 DNA Sequencer (Applied Biosystems, USA). The results were analyzed using AbiPrism DNA Sequencing Analysis version 3.7 for the Windows NT Platform (Applied Biosystems) and GeneStudio^TM^ Professional (GeneStudio, Inc., USA).

## Results

### Recovery test

Recovery tests showed that we were able to detect *E. multilocularis* DNA in samples contaminated with at least 100 eggs of this tapeworm. In comparison, the previously evaluated detection limit of nested PCR assay used in this study equaled one egg, (Szostakowska et al. 2014) which allows an estimated approximate loss of *E. multilocularis* eggs during the recovery procedure form fruit, vegetable, and mushroom samples as hundred-fold.

### Environmental contamination

In total, 103 environmental fruit, vegetable, and mushroom samples were examined with nested PCR detection method based on the *E. multilocularis* mitochondrial 12S rRNA gene. *E. multilocularis* DNA was found in 24 samples tested (23.3 %) including 12/57 samples from forests, 8/26 samples from kitchen gardens, and 4/20 samples from plantations. Positive results were recorded in all types of samples investigated including fruits, vegetables, as well as mushrooms (Table 1). Localizations of positive samples among all sampling places are shown on Fig. 1. The highest number of environmental samples contaminated with *E. multilocularis* was recorded in the districts: Ostródzki, Kętrzyński, Węgorzewski, Olsztyński, and Iławski (Fig. 1).

The number of positive samples collected from areas close to homes (kitchen gardens) were higher than those taken from remote places (forests and plantations) (30.7 and 20.78 %, respectively) (Fig. 2; Table 1).

PCR inhibition was not recorded in all negative samples tested.

The sequencing of selected positive samples and comparison with the *E. multilocularis* sequence deposited in the GenBank confirmed that the obtained PCR products represented *E. multilocularis* mitochondrial 12S rRNA gene fragments. Nucleotide sequence data reported in this paper are available in the GenBank^TM^ database under the accession numbers: KR229983, KR229984, KR229985, KR229986, KR229987, and KR229988.

## Discussion

In literature, there is no sufficient information about the occurrence of *E. multilocularis* eggs in the environment. So far, several reports described the presence of *E. multilocularis* eggs in foxes, dogs, and cat feces (Deplazes et al. 1992; Kreidl et al. 1998; Sager et al. 2006; Dyachenko et al. 2008a; Bruzinskaite et al. 2009); however, environmental matrices itself were rarely examined. Only two studies concerning detection of this tapeworm in environmental soil samples were conducted in Japan (using microscopic examination) and in Poland (using molecular assay) (Matsudo et al. 2003; Szostakowska et al. 2014). There are no reports about the distribution of *E. multilocularis* eggs on fresh fruits and vegetables intended for consumption; however, this is key information from the point of view of human health. Cool and watery fruits and vegetables present in shady forest and usually systematically watered kitchen gardens may create perfect conditions for *E. multilocularis* egg persistence. To the best of our knowledge, this is the first study confirming the presence of *E. multilocularis* eggs in environmental fruit, vegetable, and mushroom samples using molecular methods. The results of our findings provide evidence that plants may play a role in alveococcosis epidemiology. The presence of *E. multilocularis* DNA in samples examined clearly indicates that consumption of raw, unwashed fruits and vegetables and handling of fresh mushrooms may create a risk of contracting AE by humans.

We decided to use molecular methods to investigate environmental samples collected because they guarantee sensitive and specific detection of *E. multilocularis* (microscopic examination allow to detect but not differentiate of different taeniids). Previously evaluated detection limit of nested PCR assay equaled one egg (Szostakowska et al. 2014), and recovery test performed in this study showed that sample has to be contaminated with at least 100 eggs of parasite to be detectable.

*E. multilocularis* DNA was found in 23.3 % of fruit, vegetable, and mushroom samples investigated. This percentage is high, especially taking into account the imperfection of detection methods. It is not excluded that the actual level of contamination is even higher than we detected because of the fact that parasites’ eggs are dispersed widely in the environment as well as the recovery efficiency from environmental samples being limited and unsatisfactory. The detection of this parasite at low contamination of environmental sample still remains unavailable. It results mainly from the limitation of recovery procedures, during which a lot of eggs are lost. According to our experiments, flotation with saturated ZnCl_2_ solution generates hundred-fold loss of *E. multilocularis* eggs. However, using other flotation solutions, ZnSO_4_ and NaNO_3_ showed even lower recovery efficiency (data not shown).

The presence of parasites was confirmed in every type of sample tested (fruits, mushrooms, vegetables) and sampling sites (forests, plantations, kitchen gardens). *E. multilocularis* DNA was detected in a very close proportion of the samples collected from the sites away from homesteads: forests (21 %) and plantations (20 %). Whereas, contamination of samples collected close to rural homes (kitchen gardens) was visibly higher (30.7 %). It confirms our previous findings concerning contamination of soil samples collected from the territory of Varmia-Masuria Province—higher number of positive samples was also noted around homesteads (Szostakowska et al. 2014). This situation may occur, i.e., because *E. multilocularis* eggs may be dispersed more in the wide area of forests and plantations than in kitchen gardens where foxes may regularly visit the same, small area (contamination of environment may be high locally). It is significant that foxes were always seen near sampling sites. Moreover, in the past years, a significant, dynamic increase in the size of the fox population as well as *E. multilocularis* prevalence was observed in Europe, including Poland (Eckert and Deplazes 2004; Combes et al. 2012). During the last 10–15 years, the red fox population in our country increased about four times and *E. multilocularis* prevalence over three times (Karamon et al. 2014). Varmia-Masuria Province is a highly endemic region characterized by both the highest prevalence of *E. multilocularis* in the fox population (50 %) and the highest number of human AE cases in Poland. Regarding this, the number of positive samples obtained in our study is disturbing but not surprising. Foxes approach human habitats in search of food, especially in village areas. However, the results obtained might suggest that eggs were also excreted by infected dogs or cats usually present in village farmyards. This would be with accordance with many surveys showing that pets can play a role in the transmission of *E. multilocularis* to humans as they may be infected hunting for wild infected rodents (Kreidl et al. 1998; Craig et al. 2000; Kern et al. 2004; Kapel et al. 2006; Dyachenko et al. 2008b; Bruzinskaite et al. 2009; Piarroux et al. 2013). All of this creates the risk of frequent contact with a contaminated environment for humans and consequently a higher chance of contracting the disease.

Most areas investigated in this study are sparsely populated, marshy, woodland with a high population of red foxes and raccoon dogs. The samples analyzed were collected mainly from those communities where human AE cases were earlier noted: usually small villages, often poor, remote form large agglomerations, surrounded by forests and fields. Farms in which *E. multilocularis* DNA was detected, were poor, but mostly characterized by good hygienic conditions (apart from a few homesteads). People living there often eat fruits and vegetables cultivated in their own kitchen gardens rather than bought in green grocers and often earn money by picking the undergrowth (blueberries, mushrooms) in forests and trading. It shows that they often can have contact with contaminated with *E. multilocularis* eggs, plants, and fungi. The highest number of samples contaminated with *E. multilocularis* eggs were noted in the Ostródzki and Kętrzyński districts (northeast part of Varmia-Masuria Province). The results obtained from the Kętrzyński district corresponds with the data confirming not only the highest number of infected foxes (50.0–62.9 %) (Malczewski et al. 2008) but also human AE cases (15) (Nahorski et al. 2013) in this region of Poland. Taking under consideration that the period from infection time with *E. multilocularis* to first symptoms of the disease takes even up to 15 years, new AE cases may appear in the investigated area in the near future.

Forests and fields are a natural environment for foxes; therefore, it is not surprising that *E. multilocularis* DNA was detected in such locations. Among undergrowth investigated, a significantly higher number of mushroom samples were contaminated with *E. multilocularis* than berry samples (36 and 9.4 %, respectively), which is probably due to the larger and more adhesive surface of mushrooms in comparison to the small and smooth surface of forest fruits.

It is also significant that all the sequences of PCR products obtained were similar regardless of the sampling site (different districts of investigated province). Moreover, they were similar to the sequences of *E. multilocularis* isolated from soil samples in Poland collected in the same territory of Varmia-Masuria Province (Szostakowska et al. 2014), deposited in GenBank under accession number KF171965, KF1711966, KF171967 as well as to other *E. multilocularis* sequences available in the GenBank (i.e., to the isolate from *Microtus arvalis* in South Germany; L49455).

In this study, we provide evidence that fruits, vegetables, and mushrooms from endemic areas such as Varmia-Masuria Province in Poland may be contaminated with *Echinococcus multilocularis* eggs. Therefore, some measures should be undertaken to prevent infections in humans. The environment should be monitored regularly in endemic areas, and the public should be educated about this disease, its threat, and simple preventive measures such as washing hands (especially after being in contact with soil or plants) and fruits or vegetables intended for consumption as well as appropriate food handling practices (Piarroux et al. 2013).
